# A new computational model for human thyroid cancer enhances the preoperative diagnostic efficacy

**DOI:** 10.18632/oncotarget.4691

**Published:** 2015-06-29

**Authors:** Tuo Li, Jianguo Sheng, Weiqin Li, Xin Zhang, Hongyu Yu, Xueyun Chen, Jianquan Zhang, Quancai Cai, Yongquan Shi, Zhimin Liu

**Affiliations:** ^1^ Department of Endocrinology, Changzheng Hospital, Second Military Medical University, Shanghai, P. R. Chinaa; ^2^ Endocrine laboratory, Changzheng Hospital, Second Military Medical University, P. R. China; ^3^ Department of Ultrasonography, Changzheng Hospital, Second Military Medical University, P. R. China; ^4^ Department of Pathology, Changzheng Hospital, Second Military Medical University, P. R. China; ^5^ Department of General Surgery, Changzheng Hospital, Second Military Medical University, P. R. China; ^6^ Center for Clinical Epidemiology and Evidence-based Medicine, Second Military Medical University, Shanghai, P. R. China

**Keywords:** thyroid cancer, differential diagnosis, computational model

## Abstract

Considering the high rate of missed diagnosis and delayed treatments for thyroid cancer, an effective systematic model for the differential diagnosis is highly needed. Thus we analyzed the data on the clinicopathological characteristics, routine laboratory tests and imaging examinations in a cohort of 13,980 patients with thyroid cancer to establish a new diagnostic model for differentiating thyroid cancer in clinical practice. Here, we randomly selected two-thirds of the population to develop the thyroid malignancy risk scoring system (TMRS) for preoperative differentiation between thyroid cancer and benignant thyroid diseases, and then validated its differential diagnostic power in the rest one-third population. The 18 predictors finally enrolled in the TMRS included male gender, clinical manifestations (fever, neck sore, neck lump, palpitations or sweating), laboratory findings (TSH>1.56mIU/L, FT_3_>5.85pmol/L, TPOAb>14.97IU/ml, TgAb>48.00IU/ml, Tg>34.59μg/L, Ct>64.00ng/L, and CEA>0.41μg/L), and ultrasound features (tumor number≤ 23mm, site, size, echo texture, margins, and shape of neck lymphnodes). The TMRS is validated to be well-calibrated (*P* = 0.437) and excellently discriminated (AUC = 0.93, 95% CI [0.92, 0.94]), with an accuracy of 83.2%, a sensitivity of 89.3%, a specificity of 81.5%, positive and negative predictive values of 56.8% and 96.6%, positive and negative likelihood ratios of 4.83 and 0.13 in the development cohort, respectively. The TMRS highlights that this differential diagnostic system could help provide accurate preoperative risk stratification for thyroid cancer, and avoid unnecessary over- and under-treatment for such patients.

## INTRODUCTION

Thyroid neoplasm is the one of the commonest endocrine tumors worldwide with an overall malignant risk of 5~10%, and is mostly present in thyroid nodules with different pathological forms [1]. Malignant types include papillary thyroid carcinoma (PTC, 88.0%), follicular thyroid carcinoma (FTC, 5.5%), Hűrthle cell (2.3%), medullary thyroid carcinoma (MTC, 1.8%), and anaplastic thyroid carcinoma (ATC, 0.9%) [2–3]. Researchers have observed a rapid global rise in thyroid cancer incidence over the past few decades [4–6]. In developed countries, the newly diagnosed patients with thyroid cancer gradually increased from 4.9 per 100,000 in 1975 to 12.0 per 100,000 in 2011 (9.1 per 100,000 females and 2.9 per 100,000 males, respectively) [7]. It is also observed that the overwhelming rise in the incidence of thyroid cancer parallels the increasing detection rate of malignant thyroid nodules [8–9]. However, the mortality of thyroid cancer remains the same [10–12]. Therefore, some researchers propose that excessive attention to thyroid cancer may give rise to the misdiagnosis and overtreatment of thyroid cancer, which discourages the effort on the early detection [13–14].

There are various pathological types of thyroid cancers with large differences in prognosis. As National Comprehensive Cancer Network (NCCN) revealed in 2014, ATC is almost uniformly lethal, but most deaths from thyroid carcinoma occur in patients with differentiated carcinoma (e.g., PTC, FTC, or Hűrthle), which accounts for over 90% of all cases with thyroid malignancy [5]. Thus, when properly treated, most patients, especially those cases with differentiated types, can be cured or at least their life expectancy could be extended with a 5-year survival rate of 97.8% [15]. Obviously, early detection and accurate differential diagnosis are critical.

Individualized or appropriate treatment depends on the nature of the lesion. The current focus of diagnosis is to distinguish malignancies from benign growths. Fine-needle aspiration biopsy (FNAB) is the best first-line procedure for differential diagnosis of a thyroid nodule, and pathological examination is considered as the gold standard. However, up to one-third of those FNAB results are inconclusive [16–18]. Sonography is another option for screening unknown thyroid nodule and lymph node structure, but this procedure has a relatively low capacity for differential diagnosis [19]. Conventional diagnostic methods including sonography and FNAB cannot provide definitive diagnoses in many cases [20–23]. Therefore, there is an urgent need for the selection of highly accurate tests and differential diagnostic approaches to identify thyroid malignancies.

**Figure 1:**
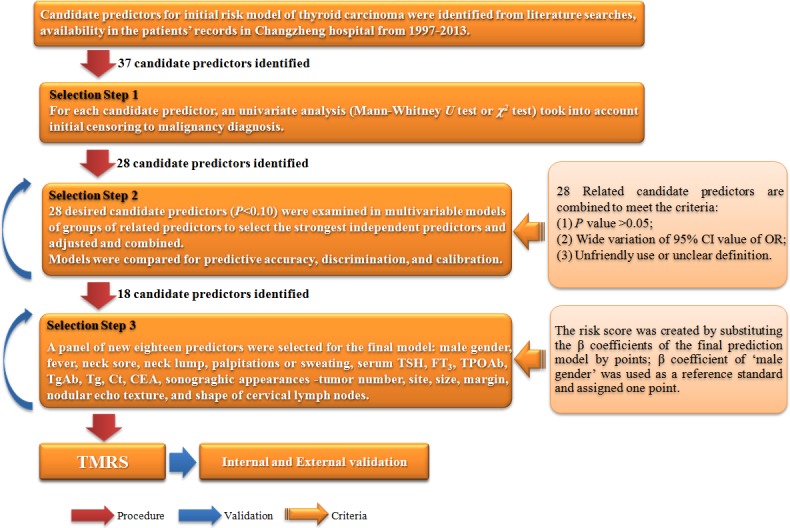
Flow chart of analytic steps in the establishment of TMRS

In the present study, we used a different computational approach to distinguish the thyroid cancer. We collected and analyzed the clinical information of nearly 14,000 patients and established a database including demographic characteristics, preoperative clinical manifestations, serological results, ultrasound results, and pathologic examination. The preoperative predictors for nodular nature were also investigated. In addition, we established and validated a risk prediction model named thyroid malignancy risk scoring system (TMRS) for the differential preoperative diagnosis for thyroid cancers (Figure 1). Our results also showed that the TMRS was a highly reliable and discriminative panel to screen predictors for thyroid cancer, and could also provide a new means of differentiating this common type of endocrine cancer.

## RESULTS

### Characterization of the patients with thyroid tumor

A total of 13,980 thyroid tumor patients with complete medical record on the preoperative examination and thyroid surgery were included.

The mean age in the study (*n* = 13,980) was 48.28 years, 77.30% were women, and 2,966 (21.22%) patients were diagnosed with thyroid carcinoma after surgery. All the participants were randomly divided into development (*n* = 9,195) and validation (*n* = 4,785) cohorts. In the development and validation cohort, the mean age was 48.75 and 48.32 years, female ratio was 77.35% and 77.26%, and 1,967 (21.39%) and 999 (20.88%) patients were diagnosed as thyroid cancer, respectively. No significant differences were found on characteristics between the two cohorts. Table 1 illustrates the detailed baseline characteristics of the patients.

**Table 1:** Baseline characteristics of the cohort by malignancy status

Characteristic	Development Cohort (*n*=9195)			Validation Cohort (*n*=4785)			Whole Cohort (*n*=13980)		
	Benign (*n*=7228)	Malignant (*n*=1967)	*P* value	Benign (*n*=3786)	Malignant (*n*=999)	*P* value	Benign (*n*=11014)	Malignant (*n*=2966)	*P* value
***Sociodemographic characteristics***									
Age (years)^a^	48.57 (12.32)	47.21 (12.29)	<0.001	48.56 (12.39)	47.41 (12.63)	0.010	48.56 (12.35)	47.28 (12.41)	<0.001
Male	1591 (22.0%)	491 (25.0%)	0.006	831 (21.9%)	257 (25.7%)	0.011	2422 (22.0%)	748 (25.2%)	<0.001
Coastal residences	2978 (41.2%)	1199 (61.0%)	<0.001	1770 (46.8%)	622 (62.3%)	<0.001	5028 (45.7%)	1821 (61.4%)	<0.001
***Symptoms & Signs***									
Fever	281 (3.9%)	22 (1.1%)	<0.001	161 (4.3%)	12 (1.2%)	<0.001	442 (4.0%)	34 (1.1%)	<0.001
Neck sore	1145 (15.8%)	150 (7.6%)	<0.001	611 (16.1%)	77 (7.7%)	<0.001	1756 (15.9%)	227 (7.7%)	<0.001
Neck lump			<0.001			<0.001			<0.001
Positive	2043 (28.3%)	420 (21.4%)		1069 (28.2%)	204 (20.4%)		3112 (28.3%)	624 (21.0%)	
Aggressive enlargement	23 (0.3%)	362 (18.4%)		9 (0.2%)	193 (19.3%)		32 (0.3%)	555 (18.7%)	
Palpations or sweating	881 (12.2%)	94 (4.8%)	<0.001	465 (12.3%)	50 (5.0%)	<0.001	1346 (12.2%)	144 (4.9%)	<0.001
Fatigue and anorexia	198 (2.7%)	48 (2.4%)	0.466	107 (2.8%)	23 (2.3%)	0.365	305 (2.8%)	71 (2.4%)	0.262
Obvious weight changes			0.134			0.160			0.026
Null	6924 (95.8%)	1903 (96.7%)		3621 (95.6%)	966 (96.7%)		10545 (95.7%)	2869 (96.7%)	
Loss	226 (3.1%)	45 (2.3%)		132 (3.5%)	23 (2.3%)		358 (3.3%)	68 (2.3%)	
Gain	78 (1.1%)	19 (1.0%)		33 (0.9%)	10 (1.0%)		111 (1.0%)	29 (1.0%)	
Dyspnea or dysphagia	77 (1.1%)	88 (4.5%)	<0.001	43 (1.1%)	48 (4.8%)	<0.001	120 (1.1%)	136 (4.6%)	<0.001
Hoarseness or dysphonia	14 (0.2%)	41 (2.1%)	<0.001	6 (0.2%)	32 (3.2%)	<0.001	20 (0.2%)	73 (2.5%)	<0.001
General malaise	87 (1.2%)	66 (3.4%)	<0.001	35 (0.9%)	27 (2.7%)	<0.001	122 (1.1%)	93 (3.1%)	<0.001
***Laboratory Findings [median (Min, Max)]***									
TSH (mIU/L)	1.56 (0.01, 11.70)	2.33 (0.01, 75.68)	<0.001	1.54 (0.01, 11.70)	2.21 (0.01, 14.05)	<0.001	1.56 (0.01, 11.70)	2.28 (0.01, 75.68)	<0.001
FT_3_ (pmol/L)	5.85 (0.80, 15.60)	6.00 (0.92, 31.95)	<0.001	5.84 (0.80, 15.60)	6.12 (1.10, 31.65)	<0.001	5.85 (0.80, 15.60)	6.06 (0.92, 31.95)	<0.001
FT_4_ (pmol/L)	19.17 (4.10, 42.0)	18.94 (1.12, 89.20)	0.939	19.20 (4.10, 37.20)	19.1 (8.14, 54.25)	0.013	19.17 (4.10, 42.00)	18.96 (1.12, 89.20)	0.168
TPOAb (IU/ml)	14.97 (0.07, 1000.00)	9.40 (0.01, 1581.00)	<0.001	15.06 (0.07, 1000.54)	10.30 (0.01, 1487.16)	<0.001	15.00 (0.07, 1000)	9.60 (0.01, 1598.81)	<0.001
TgAb (IU/ml)	48.00 (5.90, 3000)	32.18 (0.26, 7501.01)	<0.001	49.10 (5.90, 1504.30)	31.12 (0.80, 1562)	<0.001	48.40 (5.90, 3000)	31.50 (0.26, 7532.01)	<0.001
TRAb (IU/L)	0.52 (0.00, 24.50)	0.47 (0.00, 109.66)	<0.001	0.53 (0.00, 13.82)	0.49 (0.14, 106.77)	<0.001	0.52 (0.00, 24.50)	0.47 (0.09, 109.66)	<0.001
Tg (μg/L)	34.59 (0.95, 99.03)	95.00 (2.16, 334.00)	<0.001	35.22 (0.95, 99.03)	97.00 (2.16, 334.55)	<0.001	34.92 (0.95, 99.30)	96.00 (2.16, 334.00)	<0.001
Ct (ng/L)	64.00 (0.00, 121.00)	50.50 (6.02, 225.11)	<0.001	64.00 (0.10, 113.80)	52.30 (15.54, 216.60)	<0.001	64.00 (0.00, 121.0)	51.00 (6.00, 225.00)	<0.001
CEA (μg/L)	0.51 (0.00, 4.53)	0.46 (0.00, 26.30)	0.029	0.51 (0.01, 4.46)	0.45 (0.01, 26.30)	0.330	0.51 (0.00, 4.53)	0.45 (0.06, 26.30)	0.020
***Sonographic Features***									
Tumor numbers			<0.001			<0.001			<0.001
Unifocal (=1)	3532 (48.9%)	713 (36.2%)		1828 (48.3%)	369 (36.9%)		5360 (48.7%)	1082 (36.5%)	
Multifocal (>1)	3696 (51.1%)	1254 (63.8%)		1958 (51.7%)	630 (63.1%)		5654 (51.3%)	1884 (63.5%)	
Tumor site			0.008			0.023			0.001
Left lobe	2130 (29.5%)	555 (28.2%)		1032 (27.3%)	278 (27.8%)		3162 (28.7%)	833 (28.1%)	
Right lobe	2287 (31.6%)	585 (29.7%)		1291 (34.1%)	292 (29.2%)		3578 (32.5%)	877 (29.6%)	
Isthmus	57 (0.8%)	28 (1.4%)		37 (1.0%)	10 (1.0%)		94 (0.9%)	38 (1.3%)	
Both lobes	2754 (38.1%)	799 (40.6%)		1426 (37.7%)	419 (41.9%)		4180 (38.0%)	1218 (41.1%)	
Tumor size (mm)	23.28 (10.16)	18.42 (11.04)	<0.001	23.34 (10.28)	17.71 (9.64)	<0.001	23.30 (10.20)	18.18 (10.59)	<0.001
Aspect ratio (A/T)			<0.001			<0.001			<0.001
≤1	7207 (99.7%)	1643 (83.5%)		3773 (99.7%)	830 (83.1%)		10980 (99.7%)	2473 (83.4%)	
>1	21 (0.3%)	324 (16.5%)		13 (0.3%)	169 (16.9%)		34 (0.3%)	493 (16.6%)	
Calcification pattern			<0.001			<0.001			<0.001
Null	6618 (91.6%)	415 (21.1%)		3456 (91.3%)	213 (21.3%)		10074 (91.5%)	628 (21.2%)	
Microcalcification (<1mm)	36 (0.5%)	1266 (64.4%)		12 (0.3%)	628 (62.9%)		48 (0.4%)	1894 (63.9%)	
1~2mm	53 (0.7%)	156 (7.9%)		43 (1.1%)	79 (7.9%)		96 (0.9%)	235 (7.9%)	
>2mm	521 (7.2%)	130 (6.6%)		275 (7.3%)	79 (7.9%)		796 (7.2%)	209 (7.0%)	
Internal architecture			<0.001			<0.001			<0.001
Solid	4343 (68.4%)	1879 (95.5%)		2551 (67.4%)	948 (94.9%)		7494 (68.0%)	2827 (95.3%)	
Solid with cystic elements, or predominantly cystic	2285 (31.6%)	88 (4.5%)		1235 (32.6%)	51 (5.1%)		3520 (32.0%)	139 (4.7%)	
Echo texture			<0.001			<0.001			<0.001
Anechoic	40 (0.6%)	23 (1.2%)		28 (0.7%)	11 (1.1%)		68 (0.6%)	34 (1.1%)	
Hypoechoic	3189 (44.1%)	1814 (92.2%)		1695 (44.8%)	911 (91.2%)		4884 (44.3%)	2725 (91.9%)	
Isoechoic	1531 (21.2%)	85 (4.3%)		853 (22.5%)	53 (5.3%)		2384 (21.6%)	138 (4.7%)	
Hyperechoic	2468 (34.1%)	45 (2.3%)		1210 (32.0%)	24 (2.4%)		3678 (33.4%)	69 (2.3%)	
Echo pattern			<0.001			<0.001			<0.001
Homogeneous	3744 (51.8%)	67 (3.4%)		1890 (49.9%)	42 (4.2%)		5634 (51.2%)	109 (3.7%)	
Heterogeneous	3484 (48.2%)	1900 (96.6%)		1896 (50.1%)	957 (95.8%)		5380 (48.8%)	2857 (96.3%)	
Margin			<0.001			<0.001			<0.001
Well-defined	4909 (67.9%)	317 (16.1%)		2585 (68.3%)	156 (15.6%)		7494 (68.0%)	473 (15.9%)	
Ill-defined	2319 (32.1%)	1650 (83.9%)		1201 (31.7%)	843 (84.4%)		3520 (32.0%)	2493 (84.1%)	
Halo			<0.001			<0.001			<0.001
Absence	4358 (60.3%)	1862 (94.7%)		2308 (61.0%)	937 (93.8%)		6666 (60.5%)	2799 (94.4%)	
Presence	2870 (39.7%)	105 (5.3%)		1478 (39.0%)	62 (6.2%)		4348 (39.5%)	167 (5.6%)	
Posterior echo			<0.001			<0.001			<0.001
Attenuation	9 (0.1%)	456 (23.2%)		5 (0.1%)	205 (20.5%)		14 (0.1%)	661 (22.3%)	
Enhancement	22 (0.3%)	33 (1.7%)		20 (0.5%)	20 (2.0%)		42 (0.4%)	53 (1.8%)	
Neck lymph nodes shape			<0.001			<0.001			<0.001
Smooth and round	6620 (91.6%)	1372 (69.8%)		3432 (90.6%)	710 (71.1%)		10052 (91.3%)	2082 (70.2%)	
Irregular or enlarged	608 (8.4%)	595 (30.2%)		354 (9.4%)	289 (28.9%)		962 (8.7%)	884 (29.8%)	
Neck lymph nodes structure			<0.001			<0.001			<0.001
Clear	7176 (99.3%)	1531 (77.8%)		3758 (99.3%)	784 (78.5%)		10934 (99.3%)	2315 (78.1%)	
Unclear	52 (0.7%)	436 (22.2%)		28 (0.7%)	215 (21.5%)		80 (0.7%)	651 (21.9%)	
Intranodular blood-flow signal			<0.001			<0.001			<0.001
Null	641 (8.9%)	99 (5.0%)		349 (9.2%)	40 (4.0%)		990 (9.0%)	139 (4.7%)	
Hypovascular	1571 (21.7%)	604 (30.7%)		823 (21.7%)	346 (34.6%)		2394 (21.7%)	950 (32.0%)	
Hypervascular	5016 (69.4%)	1264 (64.3%)		2614 (69.0%)	613 (61.4%)		7630 (69.3%)	1877 (63.3%)	
Peripheral blood-flow signal			<0.001			<0.001			<0.001
Null	274 (3.8%)	77 (3.9%)		160 (4.2%)	31 (3.1%)		434 (3.9%)	108 (3.6%)	
Hypovascular	1521 (21.0%)	648 (32.9%)		811 (21.4%)	366 (36.6%)		2332 (21.2%)	1014 (34.2%)	
Hypervascular	5433 (75.2%)	1242 (63.1%)		2815 (74.4%)	602 (60.3%)		8248 (74.9%)	1844 (62.2%)	
Vascularity			<0.001			<0.001			<0.001
Null	2214 (30.6%)	620 (31.5%)		1172 (31.0%)	351 (35.1%)		3386 (30.7%)	971 (32.7%)	
Diffuse	5011 (69.3%)	250 (12.7%)		2613 (69.0%)	125 (12.5%)		7624 (69.2%)	375 (12.6%)	
Stripe or linear	3 (0.1%)	1097 (55.8%)		1 (0.0%)	523 (52.4%)		4 (0.1%)	1620 (54.6%)	

There are 9,195 subjects in development cohort, in which 7,228 with benignancies and 1,967 with malignancies and 4,785 in validation cohorts with Data represented as number (%) or mean (SEM), except data from laboratory findings which as median (Min, Max).

a Age and tumor size were calculated with student’s t test;

b Laboratory findings were calculated with Mann-Whitney *U* test, others were with *χ*^2^ test. There is no significant difference in characteristics among the three cohorts of the whole cohort, the development cohort, and the validation cohort.

### A panel of 28 candidate predictors for a differential diagnostic model of thyroid malignancy

**Figure 2:**
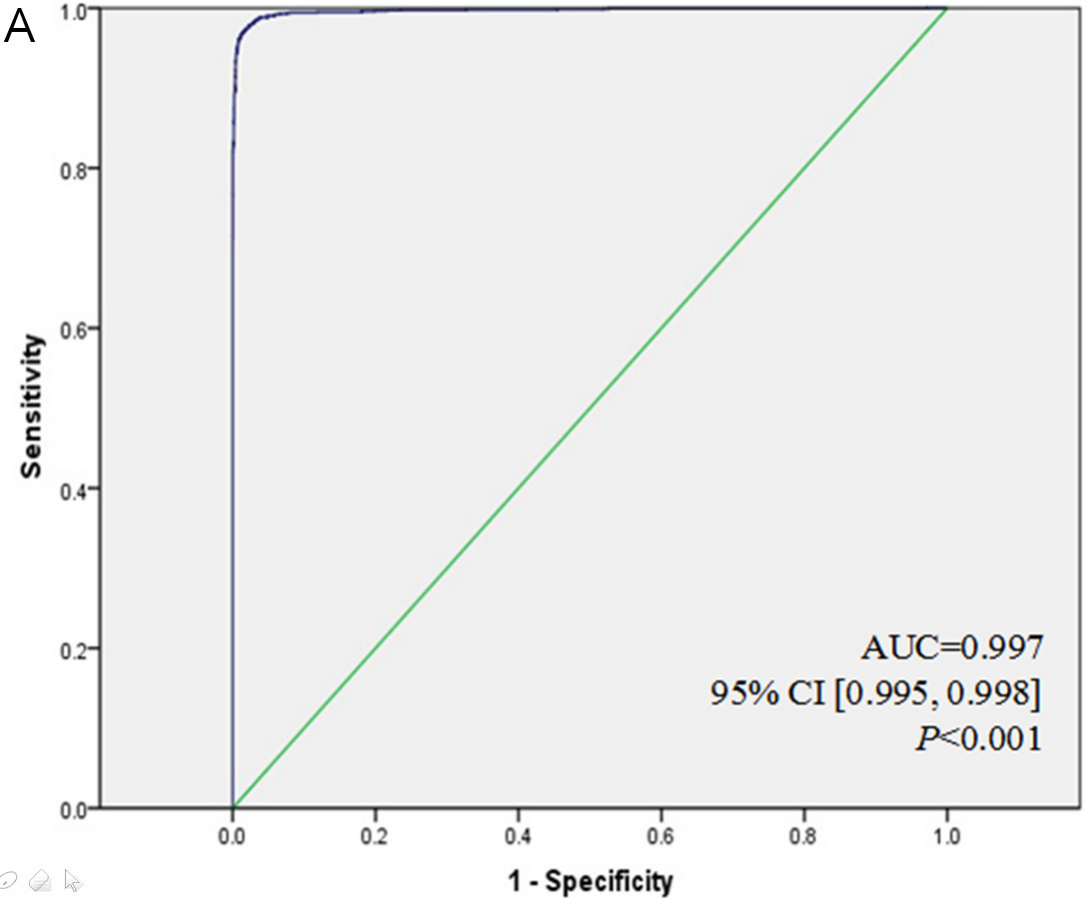

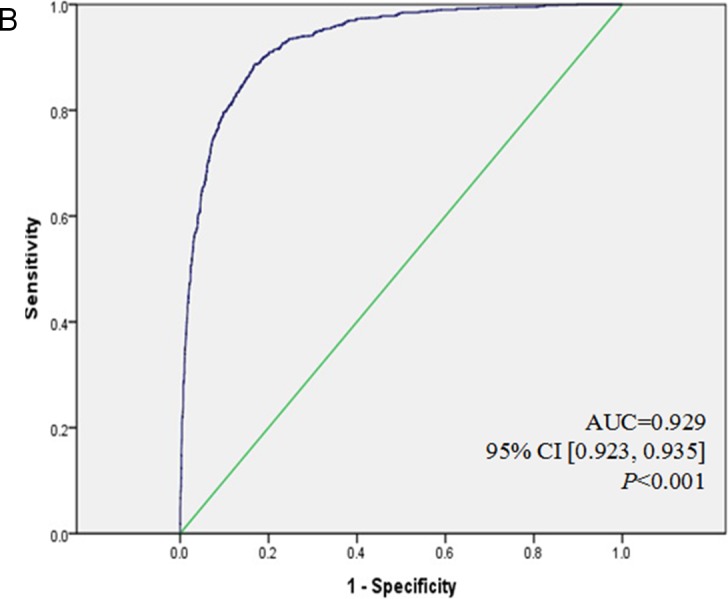

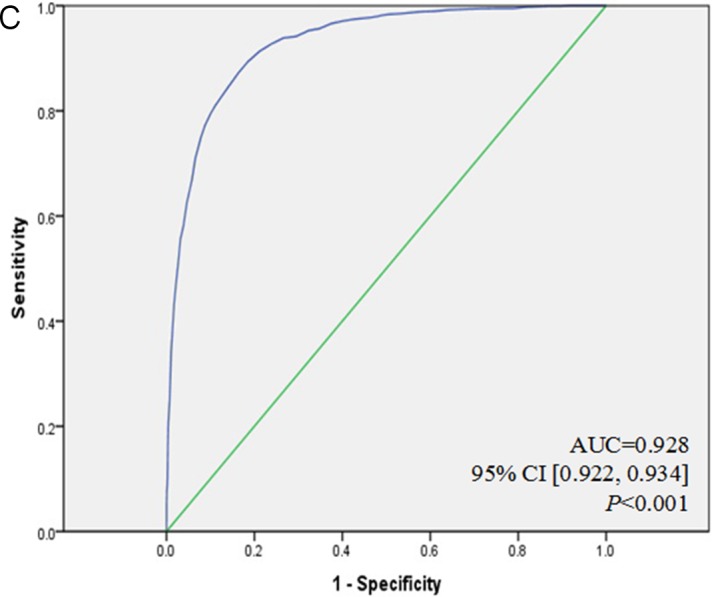

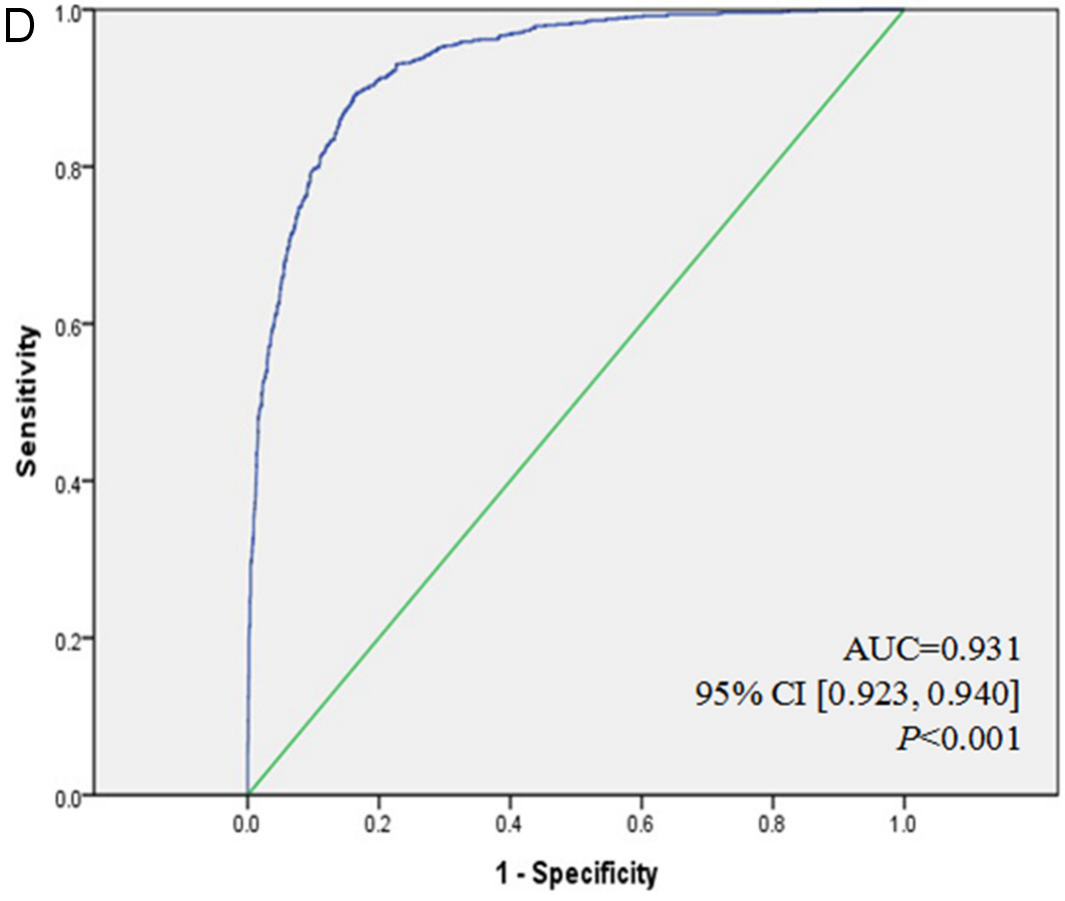
ROC curves for TMRS in development and validation cohort **A.** ROC curve for 28 candidate predictors of thyroid malignancy in the development cohort was with a very excellent discrimination (AUC = 0.997). **B.** ROC curve for the final multivariable model with 18 candidate predictors for thyroid malignancy according to Q.C.[23] and P.H.[25], still exhibited an excellent discrimination (AUC = 0.929). **C.** ROC curve for TMRS in the development cohort showed a stable and excellent discrimination (AUC = 0.928). **D.** ROC curve for TMRS in the validation cohort was with an excellent discrimination (AUC = 0.931), and there is no statistical difference between **C.** and **D.** (*P* = 0.622).

Twenty-eight of the 46 candidate predictors met the selection criteria (*P* < 0.10) for both prevalence and incidence of thyroid malignancies. The 28 selected candidate predictors were all significantly associated with diagnosis of thyroid malignancy in multiple logistic regression analysis (*P* < 0.01, Table 2), and were included in the second selection step. The receiver operating characteristic (ROC) curve showed a discrimination of the area under the ROC curve (AUC) was 0.997 (Figure 2A), demonstrating that the 28 predictors had excellent diagnostic performance. But the Hosmer-Lemeshow χ*^2^* test showed a calibration of 20.639 (*df* = 8, *P* = 0.008), indicating a significant difference between the actual and predicted malignancy diagnoses.

**Table 2:** Initial multivariable model for the thyroid malignancy with 28 predictors in the development cohort

Characteristic	Odds Ratio (OR) value	95% CI	*P* value
Gender			
Male	1		
Female	1.78	1.15, 2.77	0.010
Symptoms & Signs			
Fever			
No	1		
Yes	0.09	0.02, 0.33	<0.001
Neck Sore			
No	1		
Yes	0.11	0.06, 0.23	<0.001
Neck Lump			<0.001
No	1		
Yes	0.81	0.51, 1.28	0.361
Aggressive enlargement	231.81	69.45, 773.72	<0.001
Palpations & sweating			
No	1		
Yes	0.15	0.06, 0.35	<0.001
Dyspnea or dysphagia			
No	1		
Yes	2.94	0.98, 8.84	0.055
Laboratory Findings			
TSH (mIU/L)	1.84	1.50, 2.26	<0.001
FT3 (pmol/L)	2.18	1.83, 2.60	<0.001
TPOAb (IU/ml)	1.005	1.003, 1.006	<0.001
TgAb (IU/ml)	1.002	1.001, 1.002	<0.001
TRAb (IU/L)	1.51	1.36, 1.68	<0.001
Tg (μg/L)	1.96	1.95, 1.97	<0.001
Ct (ng/L)	2.25	1.76, 2.87	<0.001
CEA (μg/L)	1.06	1.05, 1.07	<0.001
Sonographic Features			
Tumor numbers			
Unifocal	1		
Multifocal	3.89	2.24, 6.76	<0.001
Tumor site			
Left lobe	1	0.83, 2.45	0.202
Right lobe	1.43	0.38, 13.86	0.365
Isthmus	2.30	0.19, 0.53	<0.001
Both lobes	0.32	0.92, 0.96	<0.001
Tumor size (mm)	0.94	0.92, 0.96	<0.001
A/T			
≤1	1		
>1	54.73	25.46, 117.65	<0.001
Calcifications			<0.001
Null	1		
Microcalcification (<1mm)	127.39	62.14, 261.13	<0.001
1~2mm	3.91	1.62, 9.46	0.002
>2mm	1.7	1.00, 2.91	0.051
Internal architecture			
Solid	20.41	11.05, 37.71	<0.001
Solid with cystic elements, or predominantly cystic	1		
Echo texture			<0.001
Anechoic	43.74	10.86, 176.11	<0.001
Hypoechoic	7.58	3.47, 16.56	<0.001
Isoechoic	1.32	0.51, 3.45	0.566
Hyperechoic	1		
Echo pattern			
Homogeneous	1		
Heterogeneous	12.17	6.68, 22.16	<0.001
Margin			
Well-defined	1		
Ill-defined	9.01	0.64, 82.21	0.009
Posterior echo			<0.001
Normal	1		
Attenuation	57.9	20.65, 162.35	<0.001
Enhancement	0.34	0.08, 1.43	0.141
Neck lymph nodes shape			
Smooth and round	1		
Irregular or enlarged	0.38	0.20, 0.71	0.002
Neck lymph nodes structure			
Clear	1		
unclear	6.7	2.66, 16.87	<0.001
Intranodular blood-flow signal			<0.001
Null	1		
Hypovascular	9.94	3.18, 31.05	<0.001
Hypervascular	14.24	4.05, 50.07	<0.001
Peripheral blood-flow signal			<0.001
Null	1		
Hypovascular	0.07	0.02, 0.24	<0.001
Hypervascular	0.10	0.03, 0.38	0.001

* The laboratory findings and tumor sizes are continuous variables.

### Selection of the prediction TMRS model with 18 differential diagnostic predictors

In order to improve the discrimination of the previous logistic regression model and make it convenient to use, we reduced the number of predictors in our model as much as possible, without compromising the diagnostic accuracy. Nine of the 28 candidate predictors were excluded for any of the following reasons: (1) *P* value > 0.05, (2) wide variation of 95% CI value of *OR*, or (3) difficult to use or unclear definition. We also converted all continuous variables into binary variables using the cutoff of their median values (Table 3^a^). Some candidate predictors that did not meet the criteria but were associated with thyroid malignancy were integrated into a single predictor (Table 3^b^, e.g., left lobe, right lobe and isthmus were merged into one lobe). These procedures were repeated in the second selection and logistic regression analysis to recreate and adjust the new prediction model.

**Table 3:** Final multivariable model for the thyroid malignancy risk score with 18 predictors in the development cohort

Characteristic	Odds Ratio (95% CI)	P value	β coefficient	Risk score ^c^
Gender				
Male	1.22 (1.03, 1.44)	0.024	0.197	1
Female	1			0
Symptoms & Signs				
Fever				
No	4.70 (2.82, 7.83)	<0.001	1.547	9
Yes	1			0
Neck Sore				
No	3.73 (2.96, 4.71)	<0.001	1.316	7
Yes	1			0
Neck Lump ^b^				
No	1			0
Yes	1.64 (1.40, 1.92)	<0.001	0.494	3
Palpations & sweating				
No	3.18 (2.35, 4.29)	<0.001	1.156	6
Yes	1			0
Laboratory Findings ^a^				
TSH (mIU/L)				
≤ 1.56	1			0
> 1.56	2.50 (2.09, 2.98)	<0.001	0.915	5
FT3 (pmol/L)				
≤ 5.85	1			0
> 5.85	1.66 (1.42, 1.93)	<0.001	0.505	3
TPOAb (IU/ml)				
≤ 14.97	1			0
> 14.97	2.83 (2.39, 3.36)	<0.001	1.041	6
TgAb (IU/ml)				
≤ 48.00	1			0
> 48.00	1.20 (1.02, 1.42)	0.033	0.202	1
Tg (μg/L)				
≤ 34.585	1			0
> 34.585	7.63 (6.39, 9.10)	<0.001	2.032	11
Calcitonin (ng/L)				
≤ 64.00	1			0
> 64.00	1.33 (1.13, 1.55)	<0.001	0.282	2
CEA (μg/L)				
≤ 0.41	1			0
> 0.41	1.23 (1.07, 1.43)	0.005	0.211	1
Sonographic Features				
Tumor numbers				
Unifocal	1			0
Multifocal	1.79 (1.46, 2.21)	<0.001	0.585	3
Tumor site ^b^				
One lobe	2.57 (2.11, 3.13)	<0.001	0.943	5
Both lobes	1			0
Tumor size (mm) ^a^				
≤ 23	5.07 (4.27, 6.02)	<0.001	1.623	9
> 23	1			0
Echo texture ^b^				
No or low	8.79 (7.09, 10.89)	<0.001	2.173	12
Equal or high	1			0
Margin				
Well-defined	1			0
Ill-defined	4.61 (3.89, 5.47)	<0.001	1.528	8
Neck lymph nodes shape				
Smooth and round	1			0
Irregular or enlarged	3.37 (2.75, 4.12)	<0.001	1.214	7

a Laboratory findings and tumor size were derived into binary variables by their median values;

b In the characteristic of ‘Neck lump’, aggressive enlargement was merged into Yes, ‘Tumor site’ was divided into one or both lobes according to the locations of thyroid cancer in the patient, ‘Echo texture’ was combined into two values, No or low and Equal or high;

c risk scores of each predictor were calculated by the β coefficient that they matched (i.e., predictor ‘male gender’ equals 1 point).

The new model used 18 selected candidate predictors, which were all significantly associated with differential diagnosis of thyroid malignancy in multiple logistic regression analysis. The prediction model included male gender, fever, neck sore, neck lump, palpitations or sweating, laboratory findings (TSH, FT_3_, TPOAb, TgAb, Tg, Ct, CEA), and sonographic appearances (tumor number, site, size, margin, nodular echo texture, and shape of cervical lymph nodes). This model was strongly predictive of an individual's thyroid malignancy risk, with excellent diagnostic performance (AUC = 0.929, Figure 2B) and good calibration of 7.961 (*df* = 8, *P* = 0.437).

Table 3 showed the β coefficients, odds ratio (OR), and 95% CI for the final model. Gender was used as the standard reference for assigning points for the TMRS, with the β coefficient for male gender (0.197 point) equaling one point. The points for all predictors were relative to this β coefficient (Table 3). Finally, we established this multivariable model of the TMRS and gave the involved predictors certain values (Table 3^c^), which was a scoring system with a scale from 0 to 99.

### Internal and external validation of the TMRS

**Figure 3:**
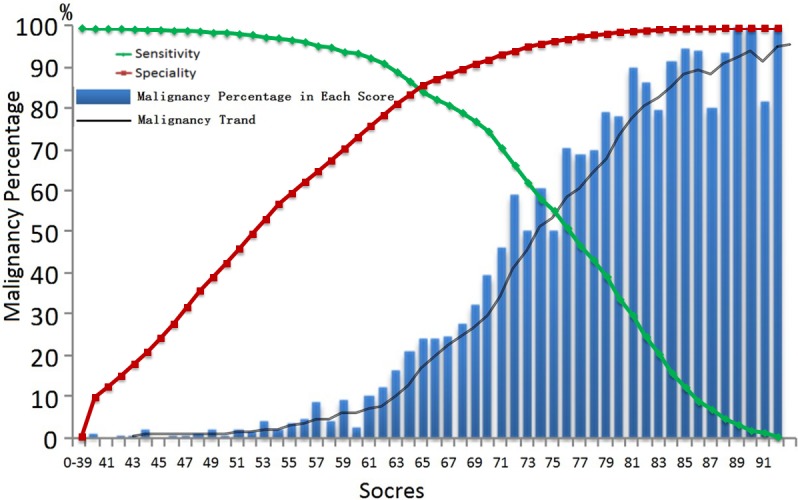

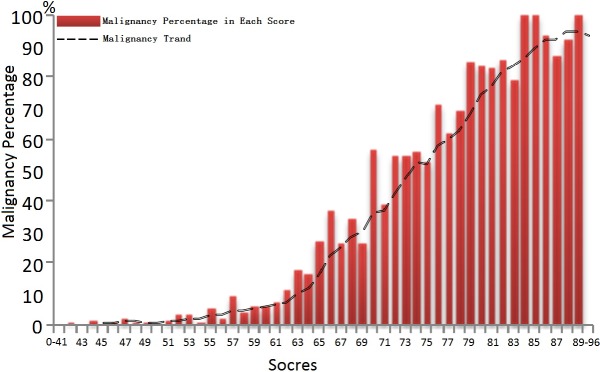
Summed score in TMRS system could predict the risk for thyroid malignancy in the development cohort (**A), the green line down and the red line up with the increasement of summed scores.At cuttoff of 65, the TMRs** boosts high sensitivity (84.5%) and high specificity (86.0%) relatively

As shown in Figure 2C, the substitution of the β coefficients with points in the TMRS only slightly decreased the AUC to 0.928. Figure 3A shows the risk of malignancy, which is reported for each summed score. The malignancy risk increased linearly with the scores. Summed scores of less than 40 (i.e., 0~39) and greater than 91 (i.e., 92~99) were fairly rare. Consequently, risk estimates and accuracy power were less stable for these score ranges. Therefore, scores ≤39 and ≥92 were collapsed into two separate categories. There was a substantial difference in thyroid malignancy risk between the lowest summed score at 39 (0%, sensitivity = 1, 1-specificity = 0.994) and the highest summed score of at least 92 (100%, sensitivity = 0.007, 1-specificity < 0.001). The detailed data were shown in Supplementary Table 1.

In the external validation cohort, all predictors in the TMRS had high differential diagnostic power in the validation cohort (*P* < 0.001, Table 4). In the validation cohort, the Hosmer-Lemeshow χ*^2^* test showed a stable calibration of 5.047 (*df* = 8, *P* = 0.753), and the AUC of 0.931 (Figure 2D). The TMRS had similar discriminations in the two cohorts (*P* = 0.622, Figure 2C *vs.* Figure 2D). Figure 3B showed the risk of malignancy, which was reported for each summed score in the validation cohort (The detailed data were shown in Supplementary Table 2).

**Table 4:** Characteristics of the external validation cohort with the diagnosis of thyroid malignancy with TMRS

Characteristic	OR value	95% CI	*P* value ^a^
Gender			
Male	1		
Female	1.64	1.29-2.09	<0.001
Symptoms & Signs			
Fever			
No	4.23	2.06-8.67	<0.001
Yes	1		
Neck Sore			
No	3.78	2.73-5.24	<0.001
Yes	1		
Neck Lump			
No	1		
Yes	1.98	1.58-2.48	<0.001
Palpations & sweating			
No	3.13	2.05-4.80	<0.001
Yes	1		
Laboratory Findings			
TSH (mIU/L)			
≤ 1.56	1		
> 1.56	3.00	2.32-3.87	<0.001
FT3 (pmol/L)			
≤ 5.85	1		
> 5.85	2.03	1.64-2.53	<0.001
TPOAb (IU/ml)			
≤ 14.97	1		
> 14.97	2.11	1.68-2.67	<0.001
TgAb (IU/ml)			
≤ 48.00	1		
> 48.00	1.73	1.37-2.18	<0.001
Tg (μg/L)			
≤ 34.585	1		
> 34.585	8.19	6.40-10.49	<0.001
Ct (ng/L)			
≤ 64.00	1		
> 64.00	1.49	1.20-1.86	<0.001
CEA (μg/L)			
≤ 0.41	1		
> 0.41	1.21	0.98-1.49	0.072
Sonographic Features			
Tumor numbers			
Unifocal	1		
Multifocal	1.57	1.17-2.11	0.003
Tumor site			
One lobe	2.53	1.91-3.34	<0.001
Both lobes	1		
Tumor size (mm)			
≤ 23	7.02	5.48-8.99	<0.001
> 23	1		
Echo texture			
No or low	8.30	6.18-11.15	<0.001
Equal or high	1		
Margin			
Well-defined	1		
Ill-defined	5.16	4.0-6.59	<0.001
Neck lymph nodes shape			
Smooth and round	1		
Irregular or enlarged	3.01	2.28-3.99	<0.001

a Calculated with χ^2^ test.

In the two cohorts, summed scores that were less than 65 (i.e., 0~64) occurred more frequently (Figure 3A and 3B). Consequently, risk estimates were more stable for these highest scores. Members with scores equal to and over 65 were considered a high risk population, with a higher malignancy detection rate (59.4% and 60.2%), while the others (0~64) were considered a low-risk population with a lower malignancy detection rate (4.0% and 3.7%). With the cutoff value of 65 points, the results of the *χ^2^* test showed that, compared with patients with the low summed scores (0~64), those with high summed scores (65~99) were 15~17 times more likely to be diagnosed with thyroid malignancy (*P* < 0.001, Table 5). The accuracy evaluations of the TMRS in development and validation cohorts were listed in Table 5. The sensitivity (SEN), specificity (SPE), accuracy, positive predictive value (PPV), negative predictive value (NPV), positive likelihood ratio (PLR), and negative likelihood ratio (NLR) of the TMRS in the development cohort were 87.0%, 83.5%, 84.5%, 59.4%, 96.0%, 5.27, and 0.16; And in the validation cohort were 87.5%, 84.8%, 85.3%, 60.2%, 96.3%, 5.76, and 0.15.

**Table 5:** Diagnostic accuracy in different risk levels of the risk score in the development and validation cohorts

Summed Scores ^a^	Development Cohort ^b^			Validation Cohort ^c^		
	Malignancy (n=1967)	Benignancy (n=7228)	Total (n=9195)	Malignancy (n=999)	Benignancy (n=3786)	Total (n=4785)
0~64	255 (4.0%)	6059 (96.0%)	6314	125 (3.7%)	3209 (96.3%)	3334
65~99	1712 (59.4%)	1169 (40.6%)	2881	874 (60.2%)	577 (39.8%)	1451

a There were 1,967 patients with thyroid malignancy and 7,228 with benignancy in the development cohort (n=9,195); 999 with malignancy and 3,786 with benignancy in the validation cohort (n=4,785).

The low risk population (0~64) and high risk population (65~99) were calculated with χ^2^ test:

b χ^2^=3608.810, df=1, P<0.001;

c χ^2^=1952.703, df=1, P<0.001.

## DISCUSSION

Among human malignancies, thyroid cancer is rare, accounting for approximately 1% of all cancers. However, it is the commonest endocrine malignancy, comprising over 90% of all endocrine cancers [24]. Early accurate detection of thyroid cancer and appropriate treatment for this disease are very important in clinical practice.

Interestingly, the Republic of Korea experienced a fifteen-fold increase in the rate of diagnosed thyroid cancer from 1993 to 2011, although the thyroid cancer mortality rate remained stable [14]. Some believe that excessive attention to thyroid cancer gives rise to overtreatment, while the other researchers suggest that the problem is not actually thyroid malignancy, but the over-diagnosis is attributable to over-screening for this type of cancer [13–14]. Welch in particular suggests that attention from the popular mass media encourages over-diagnosis and inappropriately aggressive treatment [25]. The current issue is how to best weigh the benefits of diagnosis and treatment against their harms [26].

To identify the optimal strategy for well standardized differential diagnosis for thyroid carcinoma, we evaluated the clinical significance of the specific characteristics of thyroid nodules. The TMRS presented in this study is a comprehensive analysis of an individual's absolute risk of thyroid malignancy based on a panel of predictors including thyroid-related examinations and other clinical information. The diagnostic accuracy of the TMRS was similar in the development and validation cohorts (Figure 2C *vs.* Figure 2D, Table 5). The TMRS stratifies individuals from scores of 0 to 99. Using the cut-off score of 65 (scores ≥65), the malignancy risk population is shown with a nearly 17 times difference in malignancy risk between the lower (0~64) and the higher summed scores (65~99). It performs well for all age categories and genders. For the higher risk population, they should receive further targeted FNAB with histological diagnosis or thyroid surgery. Individuals with scores < 65 appear to have lower risk of malignancy.

All markers included in the TMRS are easy to access and reading-friendly. We selected the most direct, simplest and objective measures, including demographic characteristics, clinical symptoms and serological examinations. Meanwhile, we also introduced thyroid sonography because it is widely used, but excluded some potentially confounding subjective evaluations like internal architecture, echo pattern, calcification pattern, A/T, posterior echo, neck lymph node structure, and intranodular and peripheral blood-flow signals. In contrast, some commonly used predictors were not included in the final scoring system because of their low differential diagnostic value. In this study, the identification of new predictors specific to thyroid malignancies focuses on the importance of creating a current risk score for patients with suspected thyroid nodules. More importantly, no additional technical-intensive expensive tests or invasive examinations were required, because this TMRS was mainly based on physical examinations, ultrasound imaging, FNA or other histological biopsy. A successful physical examination reveals the clinical manifestation of thyroid growths, and is a promising initiating step as effective screening method for thyroid cancer in primary care settings [1], [27]. Sonography is regarded as another optimal thyroid screening method, with lower costs and easier operation than other imaging exams [9], [19]. FNAB is the most reliable and important means for thyroid diseases worldwide, providing specimen for pathological diagnosis as the gold standard, which had been widely acknowledged by the public [28].

Our TMRS model is established by analyzing and summarizing the detailed data on preoperative clinical information including socio-demographics, clinical manifestations, serum findings and ultrasound features, and postoperative pathological diagnosis in a large diverse cohort of patients with thyroid cancers. We had the opportunity to validate the detection efficacy of this model using a large external cohort of patients, and the results exhibited a similar diagnostic accuracy of the TMRS to that in the development cohort.

Besides, the application of the TMRS may help the clinicians administrate the targeted invasive examinations or further operations for the patients with appropriate indications, which could significantly decrease the chance of over-treatment together with additional mental and economic costs. TMRS score before FNAB can prevent 66.3% of unnecessary procedure-related trauma in contrast with that by using FNAB alone. Thirty-one percentages of the population possessed high risk for malignancy. And in the other two-thirds population with lower malignant risk, about 96.5% were shown to have benign nodules after surgical treatments. The TMRS permits us to prevent as much as 84.1% of patients with benign growths from receiving excessive diagnostic procedures or treatments. On the opposite, 3.9% of patients in the low-risk cohort (TMRS < 65) had false-negative malignant nodules. A cost-benefit analysis for these patients will be conducted soon to evaluate their quality of life and economic burden.

Compared with previous studies including the thyroid nodule ultrasound forecasting model set up by Domínguez [29–31], the TMRS is the only risk score system that is specifically designed for the differential diagnosis of thyroid cancer based on comprehensive and common indicators with relatively high sensitivity and specificity. The TMRS may be useful in the selection of malignant high risk patients for early intervention especially individualized therapy in the future. Clinicians could apply this new model and scoring system to quantify the risk for malignancy, which might guide their decisions in clinical strategy and follow-up screening for such patients with thyroid cancer.

## MATERIALS AND METHODS

### Patients and study design

We identified patients with suspected thyroid tumor who were diagnosed and had surgery in Changzheng Hospital from June 1997 to December 2013, and collected their clinical information. Overall, we enrolled 13,980 cases meeting inclusion criteria. Patients who had received radiation therapy were excluded from the analysis, since radiation therapy usually interferes with the laboratory results and physical examinations. A total of 10,934 subjects were excluded from the study for any of the following reasons: (1) incomplete or missing medical records (*n* = 7,012); (2) treated thyroid tumor including local injection (*n* = 1,103), radiation therapy (*n* = 955), or thyroidectomy (*n* = 4,197) or (3) unclear treatment history (*n* = 728).

This study has been approved by the institution review board at the Second Military Medical University affiliated to Changzheng hospital. And all methods were carried out in accordance with *approved* guidelines. The informed consent was obtained from all subjects involved.

### Grouping and definition

Among all patients in the database, two-thirds (*n* = 9,195) were randomly selected for the development of the prediction model. The rest (*n* = 4,785) were used as the validation cohort. All cases were pathologically confirmed by thyroidectomy. For statistical purposes, we categorized patients with ‘Benignancy’ into thyroid Adenoma (TA), simple nodular goiter (SNG), chronic lymphocytic thyroiditis (CLT), painless thyroiditis (PPT), toxic nodular goiter (TNG), and thyroid cyst (TC). Patients with ‘Malignancy’ were categorized into PTC, FTC, MTC, ATC, uncertain malignant potential (UMP), thyroid lymphoma and other metastatic tumor.

### Socio-demographic and clinical records

The following characteristics were retrieved from the socio-demographic records: age, gender, and residence. We retrieved information regarding the clinical manifestations of thyroid conditions, including fever, neck sore, neck lump, palpitations or sweating, fatigue and anorexia, obvious weight changes (over 5 kg within 6 months), dyspnea or dysphagia, hoarseness or dysphonia, and general malaise. Serological examinations included examinations of (1) thyroid function: thyroid stimulating hormone (TSH), free three triiodothyronine (FT_3_), free thyroxine (FT_4_); (2) thyroid antibodies: thyroglobulin antibody (TgAb), thyroid peroxidase antibody (TPOAb), and thyroid stimulating hormone receptor antibody (TRAb); and (3) specific tumor biomarkers: thyroglobulin (Tg), calcitonin (Ct), and carcinoembryonic antigen (CEA).

We retrospectively reviewed the sonographic features of all cases. Real-time sonography of thyroids was performed with *Acuson Sequoia* and *128XP* sonographic scanners (Siemens Medical Solutions, Mountain View, CA), equipped with commercially available 7-MHz to 14-MHz linear probes. Color Doppler imaging and power Doppler imaging were performed with the linear array transducers. Each case was evaluated for 16 characteristics of sonography: tumor number, tumor site, tumor size, aspect ratio, calcification pattern, internal architecture, echo texture, echo pattern, margin, halo, posterior echo, shape and structure of neck lymph nodes (LN), intranodular and peripheral blood-flow signals, and vascularity. In each case, ‘tumor number’ was categorized as *unifocal* or *multifocal*, and ‘tumor site’ was categorized as *left lob*e, *right lobe, isthmus*, or *both lobes*. ‘Tumor size’ was recorded by taking the maximum value of the three diameters of anteroposterior, transverse, and vertical sections. ‘Aspect ratio’ (the anteroposterior and transverse diameter ratio, A/T) was noted as *≤1* or *> 1*. ‘Calcification pattern’ was documented in accordance with persistence and size, for instance, *null, < 1mm* (microcalcification), *1-2mm*, or *> 2mm*. ‘Internal architecture’ was defined as *solid* (cystic components > 75% of the lesion), *predominantly cystic* (> 75%), or *solid with cystic elements*. The ‘echo texture’ of each lesion was classified as *anechoic*, *hypoechoic*, *hyperechoic*, or *isoechoic* in comparison with the background thyroid tissue. ‘Echo pattern’ was divided into *homogeneous* or *heterogeneous*. ‘Margins’ of lesions were categorized as *well-defined* when lesions had clear demarcation with normal thyroid surrounding over 50% of a nodule, or *ill-defined* when > 50% of the nodular border was demarcated unclearly. The presence of a hypoechoic ‘halo’ around each lesion was also recorded as *presence* (complete) or *absence* (incomplete). ‘Posterior echo’ was grouped into *normal*, *attenuation*, or *enhancement*. Furthermore, the overall ‘shape of neck lymph node’ was classified as either *smooth and round* or *irregular or enlarged*, and ‘structure of neck lymph node’ was classified as *clear* or *unclear*. The predominant pattern of blood flow was classified as ‘intranodular blood flow’ (intrinsic to the lesion) and ‘peripheral blood flow’. Blood flow seen on color Doppler within a lesion was defined as ‘intranodular’, while flow surrounding the immediate margins of the lesion was considered ‘peripheral’. These categories were further classified as *hypovascular* or *hypervascular* with respect to lateral thyroid tissue. Additionally, ‘vascularity’ was also defined as *diffuse, striped*, or *linear*.

### Statistical analysis

We selected predictors for the prediction scoring system in three sequential steps (Figure 1). The 46 candidate predictors available in the registry and records were identified from the results of previous epidemiological and etiological studies. These predictors were evaluated against two main criteria: (1) the predictor must be significantly associated with thyroid malignancy risk in univariate analyses (continuous variables with Student's *t* test and Mann-Whitney *U* test, categorical variables with *χ^2^* test, *P* < 0.10); and (2) the remaining candidate predictors were evaluated in multivariable logistic regression models with the OR and its 95% CI (*P* < 0.05) [24]. The continuous variables in the initial model were converted into categorical variables and then repeated.

Discrimination and calibration were used to assess the predictive accuracy of the models. Discrimination refers to the model's ability to distinguish between individuals with and without thyroid malignancy, and was assessed by using the ROC curve and AUC [32–33]. Calibration refers to the agreement between predicted and actual risk, and was calculated with the Hosmer-Lemeshow *χ^2^* test [34]. Well-fitted models show non-significance on the Hosmer-Lemeshow *χ^2^* test, indicating that the modeled and actual prediction are not significantly different, and a perfect ROC test of AUC close to 1 (0.90~1 as ‘*excellent*’, 0.80~0.90 as ‘*good*’, 0.70~0.80 as ‘*fair*’, 0.60~0.70 as ‘*poor*’, and 0.50~0.60 as ‘*fail*’) [24], [33], [35].

The TMRS was created by substituting the β coefficients of each variable in the final prediction model with points. The β coefficient of male was used as a reference standard and assigned one point. ROC estimates were used to calculate the actual thyroid malignancy diagnosis per summed score. The accuracy estimates were evaluated by the SEN, SPE, accuracy, PPV, NPV, PLR, and NLR [24],[35].

For external validation, we used a cohort of the remaining 4,785 patients in the database. All of the variables in the prediction model were included in the new logistic regression model to calculate the OR value of each variable and its 95% CI. We then assessed the discrimination and calibration power of the model and its risk scoring system. Additionally, AUCs of model and risk scores in the development and validation cohorts were compared by *U* test to see whether they are consistent. With the same cutoff value, we calculated the SEN, SPE, accuracy, PPV, NPV, PLR, and NLR of the risk scoring system in the validation cohort.

We employed SPSS 19.0 Statistical Product and Service Solutions Software (release 19.0, SPSS Inc., USA) for Windows to analyze the data. Associations were judged to be significant at the 0.05 level in multivariable analyses.

## SUPPLEMENTARY MATERIAL FIGURES AND TABLES


