# Supplementary material for: A new computational model for human thyroid cancer enhances the preoperative diagnostic efficacy
Source: Oncotarget. 2015 Jun 29;6(29):28463–77. doi: 10.18632/oncotarget.4691 (PMC4695072; doi:10.18632/oncotarget.4691)
Supplement: Supplementary file 1 [file oncotarget-06-28463-s001.pdf]

A new computational model for human thyroid cancer enhances the preoperative diagnostic efficacy

Supplementary Material

Supplementary Table 1. Thyroid malignancy risk and diagnostic accuracy by each risk score in the development cohort (*n*=9195)

Summed Scores	Number at risk, *n*(%) [a]	Malignant cases, *n*(%) [b]	Sensitivity	Specificity
0-39	584 (6.35)	0 (0.0)	1.000	0.006
40	161 (1.75)	2 (1.2)	0.999	0.103
41	191 (2.08)	0 (0.0)	0.999	0.129
42	179 (1.95)	1 (0.6)	0.998	0.154
43	217 (2.36)	2 (0.9)	0.997	0.184
44	213 (2.32)	5 (2.3)	0.995	0.212
45	249 (2.71)	0 (0.0)	0.995	0.247
46	249 (2.71)	1 (0.4)	0.994	0.281
47	291 (3.16)	2 (0.7)	0.993	0.321
48	297 (3.23)	3 (1.0)	0.992	0.362
49	240 (2.61)	5 (2.1)	0.989	0.394
50	243 (2.64)	2 (0.8)	0.988	0.428
51	263 (2.86)	6 (2.3)	0.985	0.463
52	265 (2.88)	4 (1.5)	0.983	0.499
53	267 (2.90)	11 (4.1)	0.978	0.535
54	275 (2.99)	6 (2.2)	0.975	0.572
55	196 (2.13)	7 (3.6)	0.971	0.598
56	211 (2.29)	10 (4.7)	0.966	0.626
57	200 (2.88)	18 (9.0)	0.957	0.651
58	195 (2.12)	8 (4.1)	0.953	0.677
59	231 (2.51)	22 (9.5)	0.942	0.706
60	213 (2.32)	6 (2.8)	0.938	0.735
61	225 (2.45)	23 (10.2)	0.927	0.762
62	210 (2.28)	26 (12.4)	0.914	0.788
63	238 (2.59)	40 (16.8)	0.893	0.815
64	211 (2.29)	45 (21.3)	0.870	0.838
65	207 (2.25)	50 (24.2)	0.845	0.860
66	143 (1.56)	35 (24.5)	0.827	0.875
67	118 (1.28)	29 (24.6)	0.812	0.887
68	130 (1.41)	36 (27.7)	0.794	0.900
69	126 (1.37)	41 (32.5)	0.773	0.912
70	118 (1.28)	47 (39.8)	0.749	0.922
71	173 (1.88)	80 (46.2)	0.709	0.935
72	139 (1.51)	82 (59.0)	0.667	0.943

73	166 (1.81)	84 (50.6)	0.624	0.954
74	133 (1.45)	81 (60.9)	0.583	0.961
75	105 (1.14)	53 (50.5)	0.556	0.968
76	116 (1.26)	82 (70.7)	0.514	0.973
77	128 (1.39)	88 (68.8)	0.470	0.979
78	96 (1.04)	67 (69.8)	0.436	0.983
79	102 (1.11)	81 (79.4)	0.395	0.985
80	138 (1.50)	108 (78.3)	0.340	0.990
81	89 (0.97)	80 (89.9)	0.302	0.992
82	118 (1.28)	102 (86.4)	0.249	0.994
83	99 (1.08)	79 (79.8)	0.207	0.996
84	98 (1.07)	90 (91.8)	0.161	0.997
85	74 (0.80)	70 (94.6)	0.126	0.998
86	71 (0.77)	67 (94.4)	0.092	0.998
87	46 (0.50)	37 (80.4)	0.073	0.999
88	48 (0.52)	45 (93.8)	0.050	1.000
89	27 (0.44)	27 (100.0)	0.036	>0.999
90	28 (0.30)	28 (100.0)	0.022	>0.999
91	11 (0.12)	9 (81.8)	0.017	>0.999
92-99	34 (0.37)	34 (100.0)	0.007	>0.999

[a] A total of 9,195 patients were from the development cohort; [b] The percentages refer to individuals with incidence of thyroid malignancy in the summed score group.

Supplementary Table 2. Thyroid malignancy risk by each risk score in the validation cohort (*n*=4785)

Summed Scores	Number at risk, *n*(%) [a]	Malignant cases, *n*(%) [b]
0-41	456 (9.53)	0 (0.0)
42	109 (2.28)	1 (0.9)
43	101 (2.11)	0 (0.0)
44	107 (2.24)	2 (1.9)
45	133 (2.78)	0 (0.0)
46	134 (2.80)	1 (0.7)
47	141 (2.95)	3 (2.1)
48	155 (3.24)	1 (0.6)
49	157 (3.28)	1 (0.6)
50	121 (2.53)	0 (0.0)
51	131 (2.74)	2 (1.5)
52	138 (2.88)	5 (3.6)
53	161 (3.36)	6 (3.7)
54	134 (2.80)	1 (0.7)
55	111 (2.32)	6 (5.4)
56	119 (2.49)	3 (2.5)
57	119 (2.49)	11 (9.2)
58	95 (1.99)	4 (4.2)
59	112 (2.34)	7 (6.3)
60	146 (3.05)	9 (6.2)
61	97 (2.03)	7 (7.2)
62	120 (2.51)	14 (11.7)
63	117 (2.45)	21 (17.9)
64	120 (2.51)	20 (16.7)
65	81 (1.69)	22 (27.2)
66	83 (1.73)	31 (37.3)
67	56 (1.17)	15 (26.8)
68	76 (1.59)	26 (34.2)
69	76 (1.59)	20 (26.3)
70	62 (1.30)	35 (56.5)
71	87 (1.82)	34 (39.1)
72	71 (1.48)	39 (54.9)
73	71 (1.48)	39 (54.9)
74	77 (1.61)	43 (55.8)
75	68 (1.42)	36 (52.9)
76	49 (1.02)	35 (71.4)
77	63 (1.32)	39 (61.9)
78	61 (1.27)	42 (68.9)
79	60 (1.25)	51 (85.0)
80	74 (1.55)	62 (83.8)
81	52 (1.09)	43 (82.7)
82	42 (0.88)	36 (85.7)

83	48 (1.00)	38 (79.2)
84	47 (0.99)	47 (100.0)
85	34 (0.71)	34 (100.0)
86	30 (0.63)	28 (93.3)
87	23 (0.48)	20 (87.0)
88	13 (0.27)	12 (92.3)
89-96	47 (0.98)	47 (100.0)

[a] A total of 4,785 patients were from the validation cohort; [b] The percentages refer to individuals with incidence of thyroid malignancy in the summed score group.
